# Benign fibrous histiocytoma and cutaneous amyloidosis in a patient receiving enfuvirtide injections

**DOI:** 10.1016/j.jdcr.2024.04.047

**Published:** 2024-05-29

**Authors:** Ziyang Xu, Shadi Khalil, Isabelle Ponge-Wilson, Ata S. Moshiri

**Affiliations:** Ronald O. Perelman Department of Dermatology, NYU Langone Medical Center, New York, New York

**Keywords:** amyloidosis, enfuvirtide, fibrous histiocytoma, HIV

## Introduction

Enfuvirtide (Fuzeon) is a 36-amino acid biomimetic peptide strategically designed to emulate key components of the HIV fusion machinery located on the viral envelope.^1^ Working synergistically with other antiretroviral medications, it functions to impede the entry of HIV virions into host cells. Its application is particularly noteworthy in the treatment of multidrug resistant HIV strains, owing to its innovative mechanism and the relatively conserved nature of HIV fusion machineries.^2^

Although enfuvirtide is generally effective, its usage may lead to infrequent systemic adverse effects, such as hypersensitivity reactions and elevated transaminase levels in 0.1% to 1% of recipients.^3^ Notably, injection site reactions are a common occurrence, with over 80% and 70% of recipients experiencing erythema or the development of nodules/cystic lesions at injection sites respectively. However, most patients demonstrate a remarkable tolerance to these reactions.^4^

Previous studies have documented cases of enfuvirtide-induced cutaneous amyloidosis, often associated with perivascular deposits or xantho-granulomatous reactions.^5^ In this context, we present a unique case involving a 55-year-old male who had been on long-term Enfuvirtide treatment from 2014 to 2023. The patient presented with an exophytic papule on the thigh, ultimately diagnosed as enfuvirtide-induced benign fibrous histiocytoma and cutaneous amyloidosis. This case underscores the need for continued vigilance and monitoring of patients undergoing enfuvirtide therapy, considering the potential dermatologic manifestations associated with its prolonged use.

## Case report

A 55-year-old male, diagnosed with multidrug resistant HIV and currently undergoing treatment with enfuvirtide and emtricitabine/tenofovir (Descovy), presented to the dermatology clinic due to the discovery of an asymptomatic exophytic papule on his right thigh. He shared that he had initially self-administered Enfuvirtide to his abdomen, resulting in significant bruising. Subsequently, he began administering Enfuvirtide to his thighs, rotating injection sites. While these injections led to self-limited erythema and induration, he became concerned about a persistent papule on his right thigh that had been present for the past month, prompting him to seek medical attention.

**Fig 1:**
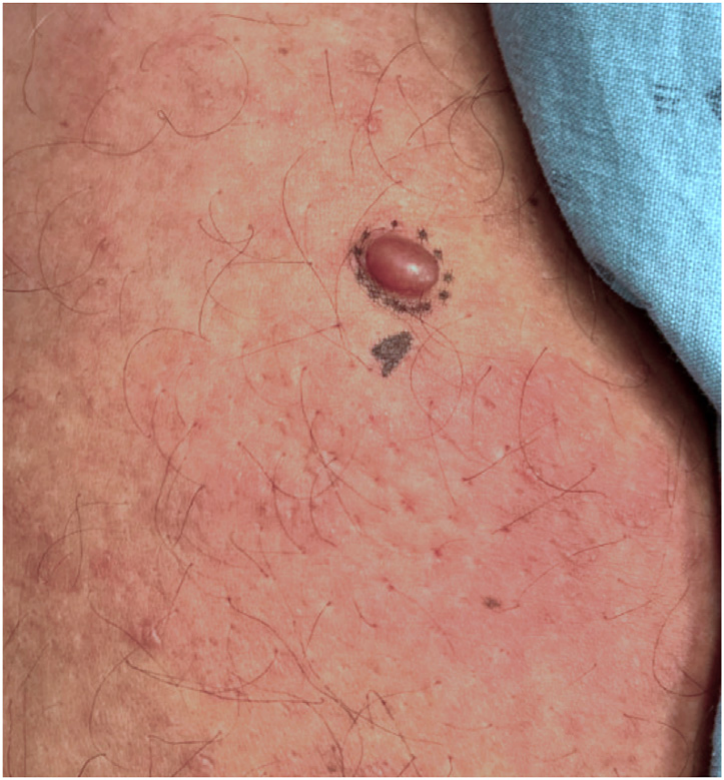
Isolated pedunculated firm skin-colored nodule arising from an erythematous indurated plaque with a peau d'orange appearance on patient’s right anterior thigh.

During the physical examination, noticeable findings included significant erythema and nodular induration covering his bilateral anterior thighs, creating a peau d'orange appearance. Specifically, there was a soft, flesh-colored exophytic papule measuring 0.8 cm × 0.8 cm × 0.3 cm on his right anterior thigh (Fig 1). In response to these clinical observations, a shave biopsy was performed.

**Fig 2:**
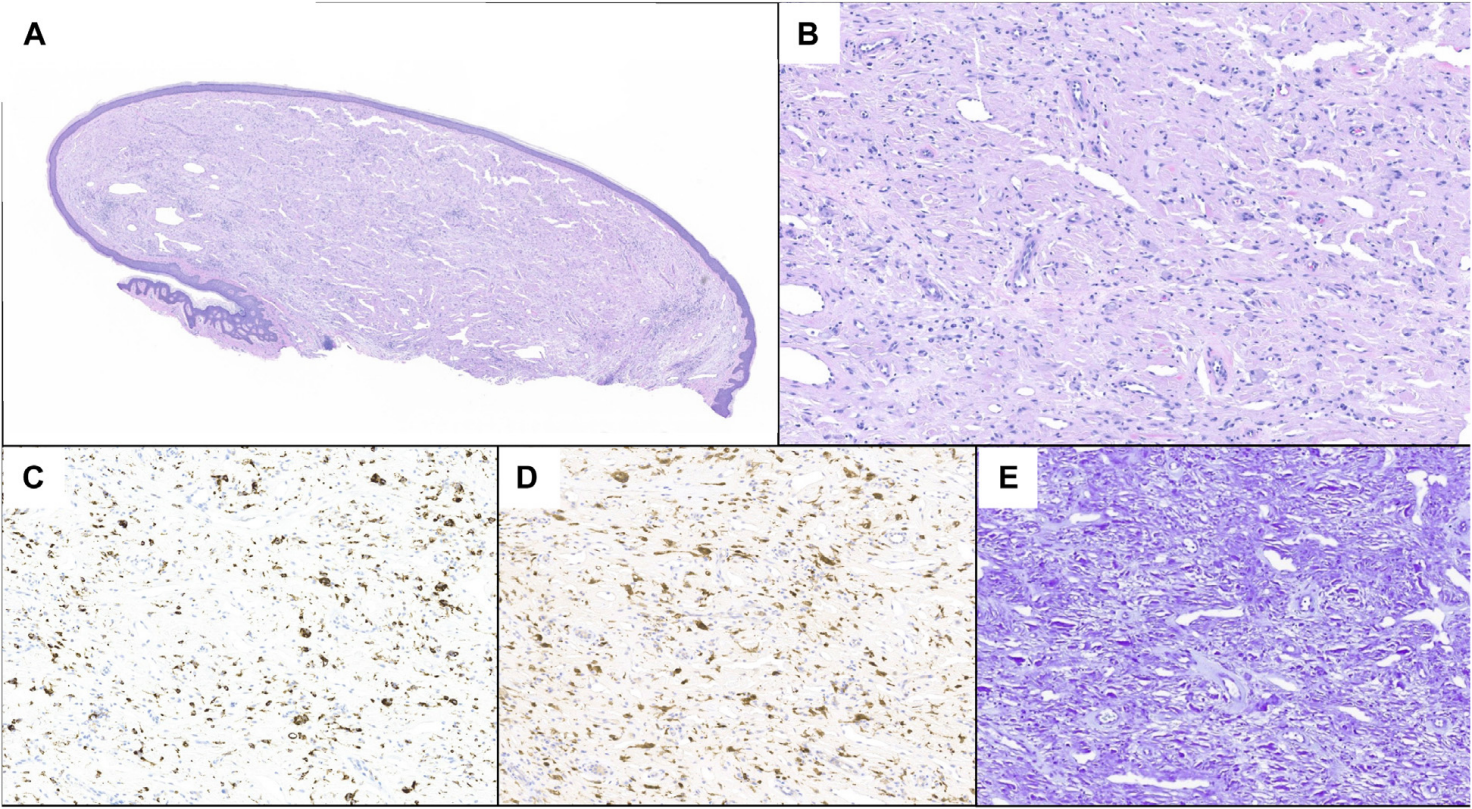
Representative histopathology. Shave biopsy of the papule demonstrated a well-circumscribed dermal proliferation (**A**) composed of spindle cells and mixed stroma by hematoxylin and eosin staining. Higher power magnification reveals a lymphoplasmacytic infiltrate with admixed stellate and occasional multinucleate cells with surrounding vessels and amorphous eosinophilic material (**B**). The spindle cells labeled positively for CD68 (**C**) and Factor XIIIa (**D**). Positive labeling of amyloid was observed following special stain with crystal violet (**E**).

Histopathological analysis was notable for a relatively well-circumscribed, bland dermal spindle cell proliferation with a surrounding epidermal collarette (Fig 2, *A*). Associated with the proliferation was an admixed lymphoplasmacytic infiltrate within a fibrovascular stroma and abundant amorphous material (Fig 2, *B*). Immunohistochemical analyses showed the spindle cells stained positively for CD68 (Fig 2, *C*) and Factor XIIIa (Fig 2, *D*) but negatively against Sox-10, CD34, HHV-8, and D2-40. Crystal violet but not Congo red or AE1/AE3 highlights amyloid deposition throughout the surrounding stroma (Fig 2, *E*), but not in the unaffected dermis. Anaplastic lymphoma kinase-staining of the specimen was negative, suggesting it may share features with other nonepithelioid fibrous histiocytomas.^6^ A diagnosis of enfuvirtide-induced benign fibrous histiocytoma and cutaneous amyloidosis was made.

## Discussion

Cutaneous amyloidosis is characterized by the extracellular deposition of amyloid protein, featuring a cross-β-pleated sheet structure, within the dermis or subcutaneous tissue.^7^ This condition can be associated with systemic amyloidosis in the context of underlying plasma cell dyscrasia (AL amyloidosis) or chronic systemic inflammatory conditions such as rheumatoid arthritis, tuberculosis, and scleroderma (AA amyloidosis).^8^ Localized cutaneous amyloidosis can result from repetitive trauma and often presents as a hyperpigmented patch overlying the scapula or may be iatrogenic, induced by subcutaneous injections of medications like enfuvirtide or insulin.^9^

Several case reports have documented enfuvirtide-induced cutaneous amyloidosis, most frequently observed in areas where injections are commonly administered, such as the abdomen and extremities (arms).^9^ A previous study employing liquid chromatography and mass spectrometry revealed that the amyloid aggregate comprised enfuvirtide peptide alongside amyloid precursor proteins like apolipoproteins A-I, A-IV, E, and serum amyloid protein.^9^ While the precise mechanism by which enfuvirtide induces amyloidosis remains unknown, it may be linked to the biomimetic peptide's inclination to aggregate. Interestingly, we found that the amyloid aggregates induced by enfuvirtide tend to be highlighted by crystal violet but not Congo red, consistent with earlier reports.^10^

In contrast to previous case reports, our patient's amyloid deposit did not incite a granulomatous reaction but was associated with a fibrous histiocytic proliferation, somewhat reminiscent of an epithelioid cell histiocytoma. This association might parallel trauma-induced fibroblast and histiocyte proliferation, akin to dermatofibroma. While these reactions are generally of low-grade severity and enfuvirtide is typically well-tolerated, future efforts to enhance the biophysical properties of fusion-peptide-based antiretrovirals could potentially reduce the incidence of injection-site reactions, representing a noteworthy avenue for translational research.

## Conflicts of interest

None disclosed.
